# Atopic Dermatitis in Italian Pre-School Children: Literature Review of Epidemiological Data with a Focus on Disease Prevalence and Severity

**DOI:** 10.3390/children10101629

**Published:** 2023-09-29

**Authors:** Iria Neri, Carlotta Galeone, Claudio Pelucchi, Gianluca Ronci, Paolo Matruglio, Maria Paola Pedone, Elena Galli

**Affiliations:** 1 Dermatology Unit, IRCCS Azienda Ospedaliero Universitaria di Bologna, University of Bologna, 40138 Bologna, Italy; 2 Department of Statistics, Bicocca Applied Statistics Center (B-ASC), Università degli Studi di Milano-Bicocca, 20126 Milan, Italy; 3 Department of Clinical Sciences and Community Health, University of Milan, 20122 Milan, Italy; 4 Sanofi S.p.A., 20158 Milan, Italy; 5 UOS Immuno-Allergologia dell’Età Evolutive, Ospedale S. Pietro-Fatebenefratelli, 00189 Rome, Italy

**Keywords:** atopic dermatitis, epidemiology, incidence, pre-school children, prevalence, review, severity

## Abstract

Atopic dermatitis (AD) is one of the most common diseases worldwide. Severe AD has a major impact on the quality of life of patients. We performed a systematic literature review on the epidemiology of AD in Italian pre-school children (age 0–5 years) and we assessed the available data on the severity of AD. In August 2022, we performed a bibliographic search using PubMed/Medline and EMBASE. We identified 10 studies with Italian data on the prevalence and/or incidence of AD in pre-school children. The period (12 months) prevalence of AD varied widely across studies, ranging between 4.0% and 42.2%, with median estimates of 14.3% among all studies and 11.8% among studies from 2010 onwards. Applied to the Italian population, this leads to a prevalence of 309,000–375,000 pre-school AD cases. Only one study computed the incidence of AD, reporting rates of 9 cases per 100 person-years in children aged 0–1 year, and 2.5 cases per 100 person-years in children aged 1–4 years. Severity data from Italy were also reviewed, across three identified three studies. A point estimate found 8.4% of cases were considered severe in one study based on the Patient-Oriented Eczema Measure (POEM), with an overall range of 7.8–11% across different Italian studies and according to various severity score types.

## 1. Introduction

Atopic dermatitis (AD) ranks 15th among the most common non-fatal diseases according to a recent report from the Global Burden of Disease (GBD) study [1]. In high-income countries, AD is estimated to affect one out of five to six children [2], with the youngest group of pre-school children (6 months to 5 years) showing the highest prevalence. In fact, the onset of AD typically occurs during the first few months of life and approximately 70% of AD cases present the first symptoms before 5 years of age [3,4]. The prevalence of disease tends to peak at around 3 years of age, after which AD may present different trajectories, with symptoms disappearing in several cases [4,5].

The same GBD study also reported that, among skin conditions, AD has the highest burden of disease measured through disability-adjusted life-years [1]. While AD is not a life-threatening condition, in some patients it presents in a severe form, affecting large body areas with intense pruritus and sleep loss. Severe disease has a significant impact on the quality of life of patients and—particularly when occurring at an early age—of parents, too [6,7]. Studies focusing on the severity of AD are relatively scanty and findings are heterogeneous between countries, showing proportions of severe cases ranging between 2% and 18% in pediatric patients and between 8% and 17% in adult patients [8,9].

During the last 25 years, several Italian investigations reporting epidemiological data on AD at the paediatric age have been published, derived from both prospective (e.g., birth cohort) studies and cross-sectional surveys [10,11,12,13]. In 2021, a review of Italian data considered the epidemiology of AD in school children and adolescents [14].

The aim of this study is, therefore, to review the available literature reporting epidemiological information on AD in pre-school children in Italy, as well as to review data on the severity of this disease in the same population.

## 2. Materials and Methods

**Figure 1:**
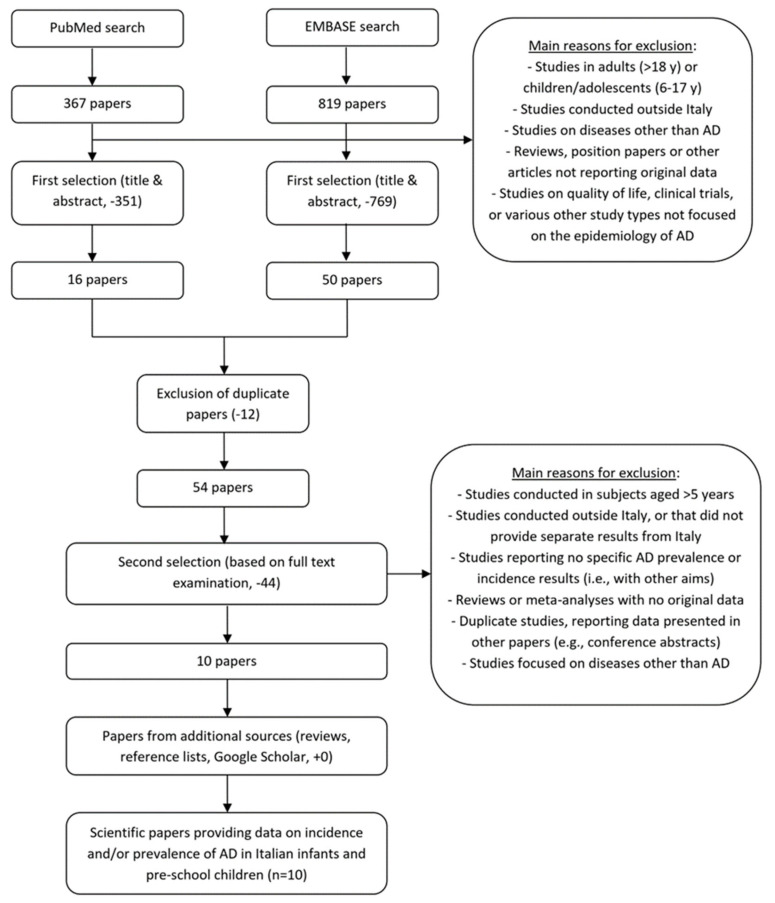
Search and selection of epidemiological studies on atopic dermatitis in Italian pre-school children.

A systematic search for studies reporting data from Italy on the prevalence and/or incidence of AD in children aged 0–5 years was conducted in August 2022, using different scientific databases and search browsers. Figure 1 presents the selection process of epidemiological studies on this topic. In PubMed/Medline, we used the following search string: “(allergic dermatitis OR atopic dermatitis OR atopic eczema OR infantile eczema OR allergic eczema) AND Italy AND (incidence OR prevalence OR epidemiology OR frequency OR occurrence OR cross-sectional OR birth cohort) AND (children OR infants)”, retrieving a total of 367 publications. The same combination of terms was applied in the EMBASE search, retrieving a total of 819 publications. No restrictions were applied according to language or publication date.

The selection process was performed by two independent reviewers. Initially, we examined the title and abstract of each identified paper and we excluded the publications that were evidently out of scope for this study’s aims. For example, we excluded studies of school children, adolescent or adult AD patients, those focused on other diseases, conducted outside Italy, that did not report original data, etc. The first step of the selection process led to the exclusion of 351 of the 367 articles identified in PubMed and of 769 of the 819 articles identified in EMBASE. In our second step, we checked the lists of publications selected in PubMed and EMBASE and we excluded duplicate ones (i.e., 12 publications were identified in both searches and were maintained after the first selection). In our third step, 54 remaining papers were examined in-depth, on the basis of their full text, to assess the potential presence of epidemiological data on AD in pre-school children in Italy. A total of 44 publications were discarded in this step, mainly because they reported results on AD related to children >5 years or to countries other than Italy, or they did not report any new/original result on the prevalence and/or incidence of AD, and 10 publications were maintained. Additional cross-validation searches for scientific articles eventually missed by the main strategy were conducted as follows: (i) by exploring the references reported in relevant reviews and epidemiological studies (i.e., those selected for inclusion in this review) of AD; and (ii) by exploring Google Scholar, using various combinations of the main search keywords (e.g., atopic dermatitis, incidence, prevalence, Italy, infants, etc.); still, no further articles relevant to the research were identified. Therefore, a total of 10 publications were included in this review of the prevalence and incidence of AD.

We extracted from each of these articles the main information on study features (e.g., study period, geographic area, sample size, study design, population, etc.), patient characteristics (e.g., age) and epidemiological findings on lifetime prevalence, period prevalence (all the articles selected considered a period of 12 months) and incidence of AD, and we summarized these data in descriptive tables in order to improve the readability and interpretation of the results.

A similar search strategy—though, in this case, not systematic—was used to identify Italian studies reporting information on the severity of AD at pre-school age, using PubMed/Medline and Google Scholar. Thus, in addition to the keywords “atopic dermatitis”, “Italy”, “children” and “infants”, we combined additional terms and scores used to address disease severity, including “severe”, “SCORAD”, “POEM” and “EASI”.

## 3. Results

Table 1 presents the main characteristics of the 10 studies included in the systematic review of epidemiological data. Three studies collected data on a national basis, while seven were based locally (three from northern, three from central Italy and one from southern Italy). Half of the studies were based on birth cohorts recruited in gynecology and obstetrics hospital departments, four were population-based cross-sectional surveys (one of them conducted via web) and one was a retrospective cohort study based on a primary care database. The diagnosis of AD in the 10 studies was based on different criteria, mainly those used in the International Study of Asthma and Allergies in Childhood (ISAAC), the UK Working Party diagnostic criteria or a clinician-made diagnosis. Four studies were focused on aims other than the incidence and/or prevalence of AD (e.g., risk factors of AD), but they still reported this information and were thus included.

The main results of the 10 studies reporting data on the prevalence of AD in pre-school children in Italy are summarized in Table 2. Most studies were based on a sample of a few hundred children; two included around 1500 subjects and one, a study from the Pedianet database of 137 pediatricians, was based on around 55,000 pre-school children. Four analyses involved children aged 0–5 years or enrolled children at birth and followed them up to 5–6 years of age; three studies considered subjects aged 3–5 years; one study followed infants during the first year of life; one followed infants from birth up to 3 years of age; and one reported results separately for children in the first year of life and from 1 to 4 years. The twelve-month prevalence of AD was examined in five papers; results varied widely across and within studies, according to year, age and type of AD diagnosis. Lifetime AD prevalence was considered in six studies. Only one study computed incidence rates of AD [15], reporting rates of 9 cases per 100 person-years in children aged < 1 year and 2.5 cases per 100 person-years in children aged 1–4 years during 2012. During the period 2006 to 2012, the incidence tended to progressively increase, as the corresponding rates were lowest in year 2006, i.e., 5 per 100 in children aged < 1 year and 1.7 per 100 person-years in children aged 1–4 years.

Table 3 summarizes the findings of studies on lifetime and period prevalence, and the incidence of AD in Italian pre-school children. Period prevalence (12 months) of AD ranged between 4% and 42%, whereas lifetime AD prevalence ranged between 8% and 34%. The corresponding median values were 14.3% and 19.3%, respectively. When only recent studies were considered, the median value of AD period prevalence was 11.8%. Further, considering the results in strata of study population, the median lifetime prevalence was 22.9% in cohorts recruited at birth in hospital and 14.5% in other population-based studies, whereas the median period prevalence was 13.3% in birth cohorts and 15.4% in other population-based studies. The only study based on primary care data reported a period prevalence of AD in around 4% in patients < 1 year and 10% in those aged 1–4 years.

Table 4 describes the main characteristics and results of three Italian studies reporting information on the severity of AD in pre-school patients. All of them were cross-sectional investigations involving patients or family paediatricians enrolled in various areas of Italy. The first study, identified through a manual (non-systematic) search, involved 437 Italian family paediatricians; overall, they reported that 23.3% of their cases had moderate AD and 7.8% had severe AD. The second study, also retrieved through a manual search, included 206 pre-school children with AD, referred to paediatric allergology or general paediatrics centres. The severity of AD was examined through the SCORing Atopic Dermatitis (SCORAD) index. Twenty-five percent of pre-school children had moderate AD (SCORAD > 25 and ≤50) and 11% had severe AD (SCORAD > 50). The third study, identified by the systematic search, was an international survey conducted in 18 countries, including Italy. Regarding the Italian data, information on the severity of disease was available for 132 children with AD diagnosed by a clinician, measured through the Patient Global Assessment (PtGA) and the Patient-Oriented Eczema Measure (POEM). According to PtGA, 34.3% of Italian cases had moderate and 10.8% had severe AD, while according to POEM, the corresponding percentages were 38.7% (for POEM 8–16) and 8.4% (for POEM > 16), respectively.

## 4. Discussion

This systematic review of national data on the epidemiology of AD in young children retrieved ten studies, mostly population-based, published between 2002 and 2021, although collecting information from the early 1990s to the late 2010s, with relevant data on this topic. The prevalence of AD measured over a 12-month period varied widely across studies, with a majority of analyses reporting disease in 10–15% of pre-school children. This is consistent with findings from international studies conducted in high-income countries [22], and higher than the national and international prevalence of AD reported in older children and in adults [8,14]. Severe disease was present in 7.8–11% of pre-school AD patients, with small differences between (a few) Italian studies.

In an earlier review of Italian epidemiological data on AD in school children and adolescents, the period prevalence, synthesized through the central (median) estimate, was 8% in both groups [14]. Using the same methods, the 12-month prevalence of AD in pre-school children computed in this review was higher, equal to 11.8% among recent studies (i.e., from 2010 onwards) and 14.3% among all studies. Applying these period prevalence estimates to the Italian population aged 0–5 years (2,621,518 children as of 1 January 2022, http://dati.istat.it, accessed on 20 July 2023), the corresponding absolute number of pre-school children with any AD—i.e., including mild, moderate and severe cases—in Italy is estimated to range between about 309,000 and 375,000 cases.

Although the majority of epidemiological information on AD at the paediatric age is focused, following the International Study of Asthma and Allergies in Childhood (ISAAC) [23,24], on children aged 6–7 and 13–14 years, a number of worldwide data are also available on the prevalence of AD in children <5 years. A recent international survey—included among the studies considered in our review—examined the issue in 18 countries, using validated methods [9]. Except for Germany, which had a low 12-month prevalence of around 7%, other European countries showed a similar or slightly higher prevalence of AD compared to Italy: 16% in the UK, 18% in France and 19% in Spain. In the USA and Canada, the prevalence in the age group 6 months to <6 years was 10% and 16%, respectively. In the US 2003 National Survey of Children’s Health, among a large representative sample of the national paediatric population, approximately 14% of children aged <4 years were reported with a diagnosis of eczema in the previous 12 months [22]. The prevalence of AD during the first year of life was 14.2% in European centres (mainly Spanish) participating in the International Study of Wheezing in Infants, which included almost 8000 subjects [25]. Therefore, our review of Italian data shows findings that are in broad agreement with those from other comparable (high-income) countries.

Recent international studies on the incidence of AD are scarce [26]. As already noted, only one such study was found in Italy, reporting incidence rates in 2012 of 2.5 per 100 person-years in children aged 1 to 4 years, and 9 per 100 person-years in children in the first year of life [15]. According to the same study, trends in incidence of AD at these ages appeared to be still increasing moderately during the period 2006–2012.

A few Italian studies reporting information on the percentage of severe AD cases in young children were identified. These used different measures and scores to classify severity of disease. This notwithstanding, their results were fairly consistent, indicating that 7.8% to 11% of Italian cases are severe at this age [9,20,21]. The international survey from Silverberg et al. was the most detailed analysis available on the topic, providing data from another 17 countries in addition to Italy, and using two different measures, i.e., PtGA and POEM [9]. POEM is a simple, patient-oriented measure, recommended by the Harmonising Outcome Measures for Eczema (HOME) Roadmap—underlying the COMET project—as the best tool to use for measuring AD symptom severity in clinical trials [27]. According to POEM, less than 13% of patients aged 6 months to 5 years had severe AD in all countries except Israel and, in most Western countries, the proportion of severe cases ranged between 5% and 12%. In Italy, the corresponding proportion according to POEM was 8.4%. Given all the above considerations, this is likely the best percentage estimate of severe AD cases in Italy. In broad agreement with recent data, an earlier study from the UK—introducing the Nottingham Eczema Severity Score—reported that 6% of AD patients in the age range 1–4 years were severe [28]. Severe AD is linked to increased morbidity and lower well-being [7,29,30]. Controlling disease severity and improving quality of life may be particularly difficult in the (small) subgroup of patients with AD that do not respond to topical steroids or other conventional therapies [31,32]. However, information to quantify the latter group in Italy is scanty.

Reviews of epidemiological data on the prevalence and incidence of disease have several limitations. In our analysis, even though it was restricted to a national setting, findings on the prevalence of AD were highly heterogeneous across studies. This is likely explained by several factors including, among others, study type (birth cohort vs. cross-sectional study), period and geographic area of investigation, age considered (e.g., infants aged 0–1 year vs. children aged 3–5 years) and, particularly, different sources and diagnostic criteria used to detect AD (e.g., reported by parents vs. diagnosed by a paediatrician or dermatologist; use of ISAAC vs. clinical criteria, etc.) [4]. Therefore, the synthesis of results is challenging. In addition to the median estimate, we presented the whole range of variation. Further, we stratified the results by period of investigation and study population, in order to identify potential sources of heterogeneity, but the study sample was limited, and most studies were population-based (i.e., nine out of ten investigations, including five studies that enrolled newborns in hospital). Given its variable course, AD is also difficult to measure epidemiologically. In fact, AD is an intermittent disease, frequently with seasonal recurrence, and may not be persistent during early childhood, as it tends to improve and disappear after age 3 in several patients. We thus considered 12-month period prevalence as a primary measure. Furthermore, data on the incidence of AD are still scanty, as we could identify only one study reporting incidence rates in Italian pre-school children [15].

In conclusion, our study confirmed and further quantified the proportion of AD cases in young children in Italy and provided an estimate of the burden of severe disease. This may be helpful to inform clinicians and health policy-makers, and thus help to plan adequate strategies to manage severe AD in pre-school children. Further data on the incidence of AD and on the percentage of young children with disease refractory to conventional treatment in Italy are needed.

## Figures and Tables

**Table 1:** Description of the studies providing information on the prevalence and incidence of atopic dermatitis (AD) in Italian pre-school children.

Source	Period	Area	Study Design	Study Population	Type of Diagnosis	Notes
Agosti et al., 2003 [10]	1994–2000	Milan-Varese	Birth cohort	Population-based (hospital newborns)	Arata Criteria	
Cantarutti et al., 2015 [15]	2006–2012	Italy	Retrospective cohort	Primary care patients	Diagnosis of AD registered by a paediatrician	
Fortes et al., 2019 [16]	NR	Rome	Birth cohort	Population-based (hospital newborns)	UK Diagnostic Criteria	Study not focused on AD incidence/prevalence, but it reports such results
Fuertes et al., 2020 [17]	2003–2008	Rome (within an international study)	Birth cohort	Population-based (hospital newborns)	Based on 2 ISAAC questions (itchy rash and localisation, in the previous 12 months)	Study not focused on AD incidence/prevalence, but it reports such results
Indinnimeo et al., 2016 [11]	NR	Rome	Cross-sectional survey	Population-based	AD in the last 12 months (ISAAC), self-reported by parents	
Parazzini et al., 2014 [12]	2007–2010	Bergamo	Birth cohort	Population-based (hospital newborns)	UK Working Party diagnostic criteria and diagnosis by a paediatrician or dermatologist, reported by parents	Study not focused on AD incidence/prevalence, but it reports such results
Parmes et al., 2020 [18]	2009–2014	Italy (within an international study)	Birth cohort	Population-based (hospital newborns)	Itchy rash that comes and goes or diagnosis of AD by a clinician	Study not focused on AD incidence/prevalence, but it reports such results
Peroni et al., 2008 [13]	NR	Verona	Cross-sectional survey	Population-based	(i) AD in the last 12 months (ISAAC), self-reported by parents (ii) AD diagnosed by a clinician	
Silverberg et al., 2021 [9]	2018–2019	Italy	Web survey	Population-based	(i) AD in the last 12 months (ISAAC) and diagnosis of AD by a clinician (ii) AD in the last 12 months (ISAAC)	
Stazi et al., 2002 [19]	1993–1994	Basilicata	Cross-sectional survey	Population-based	AD diagnosed by a clinician	

AD: atopic dermatitis; ISAAC: International Study of Asthma and Allergies in Childhood; NR: Not reported.

**Table 2:** Results of studies on the prevalence of atopic dermatitis (AD) in pre-school children in Italy.

Source	N	Age Considered	Lifetime Prevalence	Period (12 mo) Prevalence	Notes
Agosti et al., 2003 [10]	98	0–6 years	33.7% over the entire 6-year period	NR	Data for full-term children. Results of children with very low birth weight were excluded
Cantarutti et al., 2015 [15]	≈55,000 (0 y: 10,000; 1–4 y: 45,000)	(i) 0 years (ii) 1–4 years	NR	(i) From 2% (2006) to 4% (2012) (ii) From 4.5% (2006) to 10% (2012)	Year-by-year results, for the period from 2006 to 2012 Approximate results, as they were derived from two figures
Fortes et al., 2019 [16]	344	0–6 years	7.8% over the entire 6-year period	NR	Among the diagnostic criteria is ‘onset of AD before the age of 2 years’
Fuertes et al., 2020 [17]	581	4 years	NR	13.3%	Only data from the Italian GASPII cohort were reported
Indinnimeo et al., 2016 [11]	494	3–5	NR	11.8%	
Parazzini et al., 2014 [12]	796	0–1 year	- at 6 months: 17% - at 1 year: 28%	NR	
Parmes et al., 2020 [18]	274	0–3 years	17.8% over the entire 3-year period	NR	Other Italian cohorts in the study were excluded because most of the children were of school age
Peroni et al., 2008 [13]	1402	3–5	20.9% over the entire 3–5 year period (based on symptoms)	- 18.1% on the basis of symptoms - 15.4% on the basis of a clinician’s diagnosis	
Silverberg et al., 2021 [9]	1547	6 months to <6 years	NR	- 15.3% on the basis of symptoms and clinical diagnosis - 42.2% on the basis of symptoms alone	The study reports information on the severity of AD, based on PGA and POEM
Stazi et al., 2002 [19]	201	3 months to 5 years	8.0%	NR	

AD: atopic dermatitis; NR: not reported; PGA: Patient Global Assessment; POEM: Patient-Oriented Eczema Measure.

**Table 3:** Summary of results of studies on atopic dermatitis (AD) in Italian pre-school children (0–5 years) ^a^.

	All Studies				Only Recent Studies (≥2010)			
Epidemiological Measurement	No. of Studies	No. of Measures	Range	Median	No. of Studies	No. of Measures	Range	Median
Lifetime prevalence	6	6	7.8–33.7%	19.3%	2	2	7.8–17.8%	12.8%
Period prevalence, 12 months	5	8 ^b^	4.0–42.2% ^b^	14.3%	3	5 ^b^	4.0–42.2% ^b^	11.8%
Incidence rate (/100 py)	1	2 ^b^	2.5–9.0 ^b^	5.7	1	2 ^b^	2.5–9.0 ^b^	5.7

^a^ The sum of the studies exceeds the total of 10 because some studies reported more than one epidemiological measure. ^b^ For the study from Cantarutti et al., 2015 [15], that provided yearly incidence and prevalence estimates from 2006 to 2012, only the latter (most recent) estimate was included.

**Table 4:** Results of Italian studies with information on the proportion of severe cases of atopic dermatitis (AD) in patients aged 0–5 years.

Source	Area	Study Design	No. Enrolled	Severity Measurement	Indicator/Level	Result
Ruggiero et al., 2012 [20]	Italy, 16 regions involved	Survey of family paediatricians	437	Simple question on the case history of each paediatrician	% of cases evaluated as:	
					(1) Mild	68.9%
					(2) Moderate	23.3%
					(3) Severe	7.8%
Galli et al., 2020 [21]	Italy, 9 cities	Cross-sectional, paediatric or allergy centre patients	206 at pre-school age	SCORAD	% of cases with SCORAD:	
					(1) ≤25 (mild)	64%
					(2) 26–50 (moderate)	25%
					(3) >50 (severe)	11%
Silverberg et al., 2021 [9]	Italy (within an international study conducted in 18 countries)	Cross-sectional, web-survey	1547, of which 132 with AD diagnosed by a clinician	POEM and PtGA	% of cases with POEM:	
					(1) 0–7 (mild)	52.2%
					(2) 8–16 (moderate)	38.7%
					(3) >16 (severe)	8.4%
					% of cases with PtGA:	
					(1) ≤1 (null/mild)	54.2%
					(2) 2 (moderate)	34.3%
					(3) 3 (severe)	10.8%

AD: atopic dermatitis; POEM: Patient-Oriented Eczema Measure; PtGA: Patient Global Assessment.

## Data Availability

No new data were created or analyzed in this study. Data sharing is not applicable to this article.
